# CEP120 interacts with C2CD3 and Talpid3 and is required for centriole appendage assembly and ciliogenesis

**DOI:** 10.1038/s41598-019-42577-0

**Published:** 2019-04-15

**Authors:** Jhih-Jie Tsai, Wen-Bin Hsu, Jia-Hua Liu, Ching-Wen Chang, Tang K. Tang

**Affiliations:** Institute of Biomedical Sciences, Academia Sinica, Taipei, Taiwan

**Keywords:** Cilia, Centrosome

## Abstract

Centrosomal protein 120 (CEP120) was originally identified as a daughter centriole-enriched protein that participates in centriole elongation. Recent studies showed that *CEP120* gene mutations cause complex ciliopathy phenotypes in humans, including Joubert syndrome and Jeune asphyxiating thoracic dystrophy, suggesting that CEP120 plays an additional role in ciliogenesis. To investigate the potential roles of CEP120 in centriole elongation and cilia formation, we knocked out the *CEP120* gene in p53-deficient RPE1 cells using the CRISPR/Cas9 editing system, and performed various analyses. We herein report that loss of CEP120 produces short centrioles with no apparent distal and subdistal appendages. CEP120 knockout was also associated with defective centriole elongation, impaired recruitment of C2CD3 and Talpid3 to the distal ends of centrioles, and consequent defects in centriole appendage assembly and cilia formation. Interestingly, wild-type CEP120 interacts with C2CD3 and Talpid3, whereas a disease-associated CEP120 mutant (I975S) has a low affinity for C2CD3 binding and perturbs cilia assembly. Together, our findings reveal a novel role of CEP120 in ciliogenesis by showing that it interacts with C2CD3 and Talpid3 to assemble centriole appendages and by illuminating the molecular mechanism through which the CEP120 (I975S) mutation causes complex ciliopathies.

## Introduction

Centrioles are microtubule (MT)-based organelles that exist as part of the centrosome and are also essential for the formation of cell structures known as cilia and flagella. Centriole duplication starts at the G1-S transition of the cell cycle. A nascent centriole (daughter centriole) starts to grow from the proximal end of a pre-existing centriole (mother centriole) in G1 phase of cell cycle, elongates through the S and G2 phases, and reaches its full length at early mitosis. In this cell cycle, the daughter centriole has not yet acquired subdistal appendages (SDAs) and distal appendages (DAs), cannot be basal body for primary cilia, but can produce new daughter centriole near the proximal end^1–5^. After its second round of mitosis, this centriole becomes a fully mature mother centriole^1–5^.

The primary cilium is a hair-like MT-based structure that protrudes from the cell surface and is responsible for sensing extracellular signals and regulating cell homeostasis and development. We previously showed that myosin-Va mediates the transportation of preciliary vesicles (PCVs) to the DA of a mother centriole as the earliest event that defines the onset of ciliogenesis^6^. These PCVs fuse with other incoming PCVs to form a large ciliary vesicle through the membrane shaping proteins EHD1 and EHD3^7^. This initial step that converts the mother centriole into a basal body, is followed by removal of the centriolar coiled coil protein 110 (CP110) that caps the distal end of the mother centriole, the assembly of ciliary transition zone, and the growth of axoneme microtubules during ciliogenesis^8^.

A current model holds that centriole duplication occurs after activation of PLK4, followed by assembling a SAS-6-containing cartwheel at the proximal end of mother centriole^9–14^. CEP135 serves as a bridge molecule that connects SAS-6 and CPAP, linking the cartwheel to the centriolar MTs^15^. CPAP then acts together with CEP120 and SPICE to promote the assembly and elongation of centriolar MTs during S/G2 phase^16,17^. However, we do not yet fully understand the molecular basis through which a full-length centriole assembles its own SDAs and DAs to become a mature centriole and how a mature centriole is converted to a basal body to initiate ciliogenesis. *C2CD3* and *Talpid3* are evolutionarily conserved genes essential for vertebrate development and ciliogenesis^18–22^. Recent studies showed that C2CD3 is required for the recruitment of centriolar DA proteins^21^ and loss of C2CD3 results in the shortening of centrioles and the loss of DAs and SDAs^22^. Talpid3 was previously reported to be essential for ciliary vesicle docking during ciliogenesis^20^. A very recent report showed that Talpid3 is physically associated with C2CD3 at the distal ends of centrioles and such an association is essential for centriole maturation and DA assembly^23^.

Our group and others previously showed that centrosomal protein 120 (CEP120) is a daughter centriole-enriched protein that plays essential roles in centriole duplication, elongation, and maturation^16,17,24,25^. Interestingly, mutations in the *CEP120* gene lead to severe human genetic diseases, including Jeune asphyxiating thoracic dystrophy (JATD) and Joubert syndrome (JS), both of which involve complex ciliopathy phenotypes^26,27^. To date, nine mutations have been identified in the *CEP120* gene^26,27^. Among them, the missense mutations V194A and A199P, which cause JS and JATD, respectively, were recently reported to reduce CEP120 protein levels and impair cilia formation^28^. Furthermore, inactivation of *Cep120* in the central nervous system of mice was found to cause severe cerebellar hypoplasia and loss of cilia on ependymal cells^29^. However, we have an incomplete understanding of the clinical relevance of *CEP120* mutations or the molecular mechanisms through which they cause defects in centriole elongation and cilia formation.

Here, we report that complete loss of CEP120 is associated with the production of short centrioles and the absence of functional DAs and SDAs at the mother centriole. Mechanistically, we show that CEP120 is essential for the recruitment of C2CD3 and Talpid3 to the nascent centrioles, and is responsible for promoting the assembly of the centriole appendages that are needed for subsequent ciliogenesis.

## Results

### Loss of CEP120 produces short centrioles with no apparent DA and SDA structures

We and others previously showed that CEP120 cooperates with CPAP to regulate centriole elongation^16,17^. However, it was unclear how CEP120 regulates centriole elongation. To address this question, we herein used the CRISPR/Cas9-mediated gene editing system^30^ to generate stable hTERT-RPE1 cell lines that harbor inactivating mutations in both *CEP120* and *p53* (*CEP120−/−; p53−/−*). The *p53* gene mutation was introduced because cells without centrioles seem to be non-viable in the presence of p53^31^ and loss of p53 was reported to have no effect on centriole elongation^32^. We obtained two independent CEP120-knockout cell lines (KO-1 and KO-2) and their sequences were confirmed (Supplementary Fig. S1a). Immunofluorescence analysis (Fig. S1b) and Western blotting (Fig. S1c) revealed no detectable CEP120 signal.

**Figure 1:**
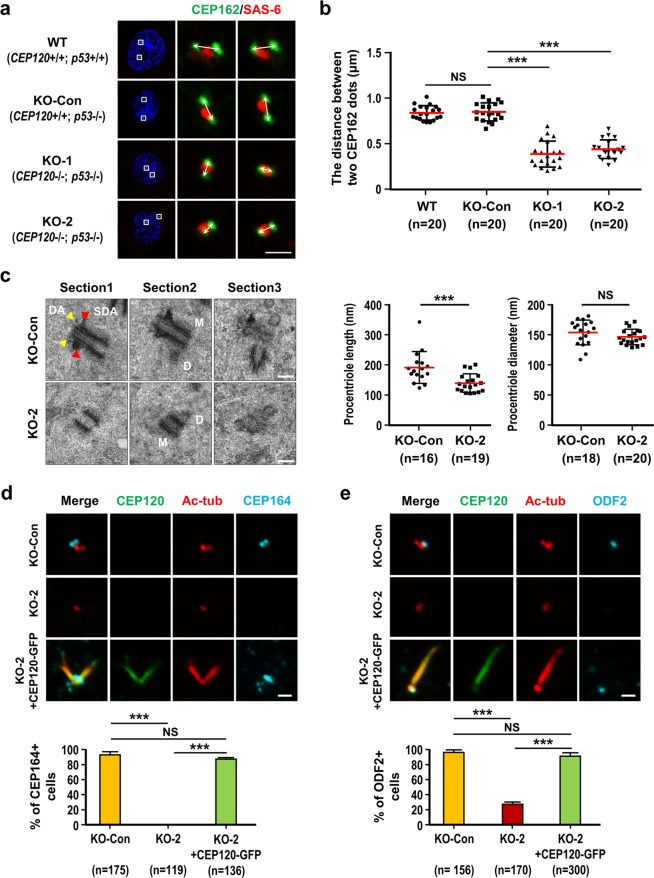
Loss of CEP120 produces short centrioles with no apparent distal appendage (DA) and subdistal appendage (SDA) structures. RPE1-based wild-type cells (WT; *CEP120*+/+; *p53*+/+), control cells (KO-Con; *CEP120*+/+*; p53−/−*), and knockout cell lines KO-1 (*CEP120−/−; p53−/−*, clone #3-6-3) and KO-2 (*CEP120−/−*; *p53−/−*, clone #34) were treated with aphidicolin for 24 h and released in fresh medium for another 15 h to allow progression to G2 phase. (**a)** Cells were examined by confocal immunofluorescence microscopy using the indicated antibodies. (**b)** Histogram illustrating the distances between two CEP162 dots. (**c)** Representative images of three serial electron microscopy (EM) sections (100 nm/each) of the same centrioles in KO-Con and KO-2 cells. The distal appendages (DA, yellow arrowheads) and subdistal appendages (SDA, red arrowheads) are seen in the mother centriole of KO-Con cells but are absent from KO-2 cells (M: mother; D: daughter). Histogram illustrating the length and diameter of procentrioles in KO-Con and KO-2 cells, as analyzed by EM. Error bars in (**b**,**c**) represent the mean ± s.d.; ***P < 0.001; NS, not significant. (**d**,**e**) Rescue experiments. KO-Con, KO-2, and KO-2 cells expressing doxycycline-inducible CEP120-GFP WT cells were synchronized at G2, fixed, and stained with the indicated antibodies. Histogram illustrating the percentages of CEP164-positive cells (**d**, lower panel) or ODF2-positive cells (**e**, lower panel). Error bars represent the mean ± s.d. from pools of cells (n) from three independent experiments. ***P < 0.001; NS, not significant. Note that overexpression of CEP120-GFP induces centriole over-elongation. Scale bars, 1 μm in (**a**,**d**,**e**) 100 nm in (**c**).

To examine whether CEP120 regulates centriole elongation, cells were synchronized at G2 phase; this phase was chosen because the duplicated centrioles could be clearly distinguished from one another and the procentriole was at nearly its full length. The cells were stained with antibodies against CEP162 (a distal-end marker)^33^ and SAS-6 (a marker of newborn centriole)^34^. We measured the distance between two CEP162 dots^32,35^ and found that the mean distance between two CEP162 dots in KO-1 and KO-2 cells was significantly shorter than that in wild-type (WT) and p53-KO-Control cells (hereafter referred to KO-Con), and that there was no significant difference in the length of WT and KO-Con cells (Fig. 1a,b). Electron microscopy (EM) revealed that the average length of the nascent centriole (G2 phase) in KO-2 cells was significantly shorter than that in KO-Con cells (Fig. 1c, middle panel), whereas there was no significant difference in the diameter of the nascent centriole at G2 phase (Fig. 1c, right panel).

To examine whether the CEP120 loss-induced short centrioles could also be found in p53 WT cells, we depleted CEP120 in RPE1 (p53 WT) and RPE1-based PLK4-myc-inducible cells. Both control (siControl) and CEP120-depleted (siCEP120) cells were synchronized at G2 and analyzed by confocal immunofluorescence microscopy (for details, see Fig. S1a). We found that the mean distance between two CEP162 dots was significantly decreased in siCEP120-treated cells comparing to the control cells (Fig. S2b). In consistent with this finding, we frequently observed multiple shortened G2 centrioles in siCEP120-treated PLK4-myc-inducible cells (Fig. S2c). A similar finding was also observed by Comartin *et al*.^16^.

Unexpectedly, images obtained from series-section EM showed that the complete loss of CEP120 produced shortened centrioles that lacked apparent DAs and SDAs (KO-2 cells, Fig. 1c). This phenotype was not commonly observed in siCEP120-treated cells^16^, probably due to the expression of a residual amount of CEP120 that allowed the formation of DAs and SDAs. Further immunofluorescence analysis showed that dramatically fewer KO-2 cells were positive for CEP164 (a known DA marker)^36^ and ODF2 (a SDA marker)^37^, and that both signals could be effectively rescued by exogenous expression of GFP-tagged full-length CEP120-GFP (Fig. 1d,e). Similar results were also observed when we used CEP83 (Fig. S3a) and CEP128 (Fig. S3b) as alternative markers for DAs and SDAs^38,39^. We previously reported that CEP120 overexpression could induce overly long centrioles and have no obvious effect on the formation of DAs and SDAs^17^. As noted in the rescue experiments (Fig. 1d,e), overly long centrioles were frequently observed in CEP120 overexpressing cells (Fig. 1d,e). Based on the above findings, we conclude that loss of CEP120 produces short centrioles and impairs DA and SDA assembly, and these effects can be effectively rescued by exogenous expression of CEP120.

### Loss of CEP120 yields a defect in centriole elongation

Since the loss of CEP120 led to the production of shortened centrioles, we examined how the loss of CEP120 affects centriole elongation. It was reported that depletion of CEP120 has no effect on initial cartwheel assembly^16^. Consistent with this finding, our *CEP120* gene knockout cells (KO-2) did not reveal any change in the recruitment of SAS-6 (Fig. S3c) and STIL (Fig. S3d), two known early-born centriolar proteins, to the procentrioles during early S phase.

**Figure 2:**
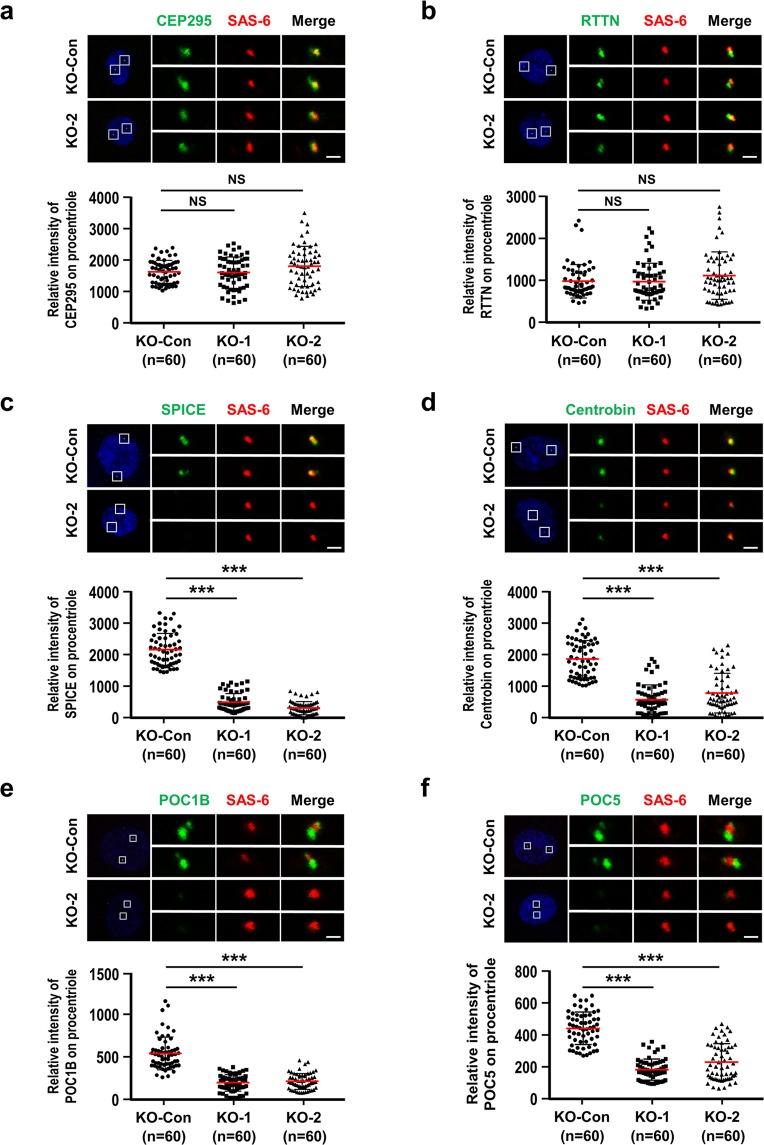
CEP120 loss shows a defect in centriole elongation. (**a**–**f**) KO-Con, KO-1, and KO-2 cells were synchronized at G2 phase and analyzed by immunofluorescence confocal microscopy using antibodies against CEP295 (**a**), RTTN (**b**), SPICE (**c**), centrobin (**d**), POC1B (**e**), POC5 (**f**), and SAS-6 (**a**–**f**). Histogram illustrating the relative intensities of the CEP295 (**a**), RTTN (**b**), SPICE (**c**), centrobin (**d**), POC1B (**e**), and POC5 (**f**) spots on procentrioles. Error bars represent the mean ± s.d. ***P < 0.001; NS, not significant. Scale bar, 1 μm.

We further investigated whether the loss of CEP120 affected the localization of several known centriole elongation-related proteins, including CEP295^40^, RTTN^32^, SPICE^16^, and centrobin^41^, and the later-born centriolar proteins, POC1B^42^ and POC5^35^, to the new centrioles in G2 phase. *CEP120* knockout (KO-1, KO-2) and KO-Con cells were synchronized at G2 and immunostained with the indicated antibodies. As shown in Fig. 2, loss of CEP120 had no apparent effect on the recruitment of the centriolar inner lumen proteins, CEP295 (Fig. 2a) or RTTN (Fig. 2b), to the CEP120-KO centrioles, but the localization of SPICE (Fig. 2c) and centrobin (Fig. 2d) to the CEP120-KO centrioles was greatly reduced. Furthermore, as expected, loss of CEP120 significantly inhibited the loading of POC1B (Fig. 2e) and POC5 (Fig. 2f) to the distal part of the centriole inner lumen. Together, our results indicate that complete loss of CEP120 produces short centrioles with a defect in centriole elongation, and that although initial cartwheel formation proceeds normally, there are alterations in the recruitment of several centriole elongation proteins (SPICE and centrobin) and two later-born proteins (POC1B and POC5) to the G2-nascent centrioles.

### Loss of CEP120 impairs the recruitment of C2CD3 and Talpid3, but not CP110, to the distal ends of centrioles

**Figure 3:**
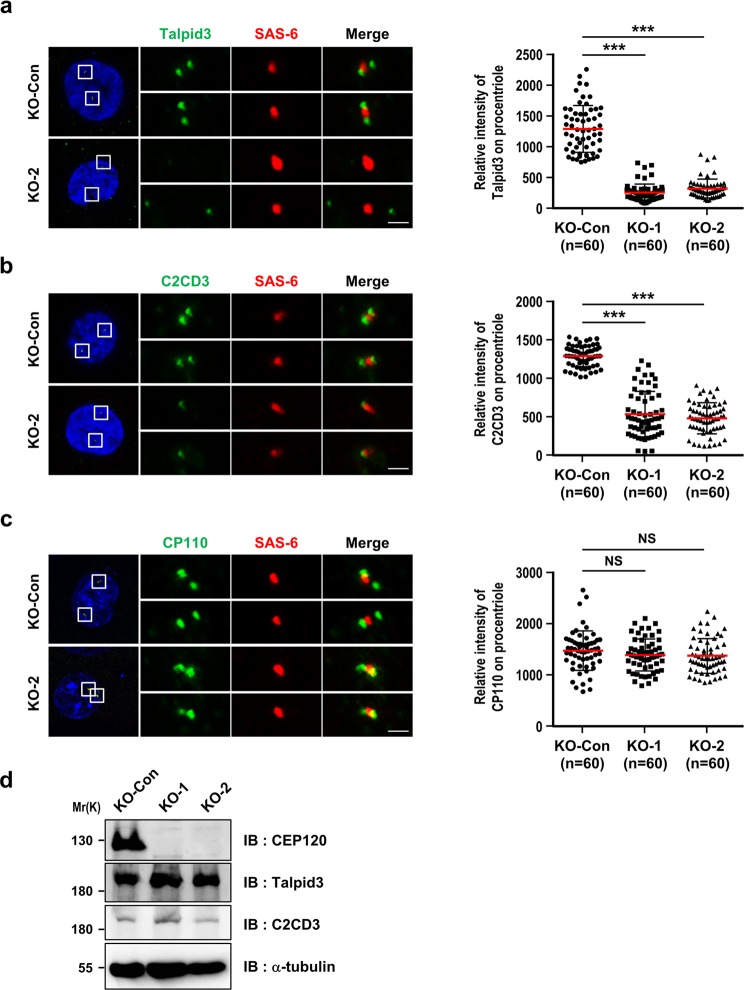
CEP120 loss shows defective recruitment of the centriolar distal-end proteins C2CD3 and Talpid3. (**a**–**c**) KO-Con, KO-1, and KO-2 cells were synchronized at G2 phase and analyzed by immunofluorescence confocal microscopy using antibodies against Talpid3 (**a**), C2CD3 (**b**), CP110 (**c**), and SAS-6 (**a**–**c**), and the results were quantified. Histogram illustrating the relative intensity of Talpid3 (**a**), C2CD3 (**b**), and CP110 (**c**) on procentrioles. (**d**) The protein expression levels of endogenous CEP120, Talpid3 and C2CD3 were examined by immunoblotting using the indicated antibodies. Uncropped blots are shown in Fig. S6a. Error bars represent the mean ± s.d. ***P < 0.001; NS, not significant. Scale bar, 1 μm.

Recent reports indicate that a number of centriole distal-end proteins, including C2CD3^21,22^, Talpid3^20^, and CP110^20,43–46^, involved in regulating centriole elongation, DA and SDA assembly, and ciliogenesis, and that mutations of these genes cause ciliogenic defects. Accordingly, we next used confocal fluorescent microscopy to examine whether the loss of CEP120 affected the localization of these known distal-end proteins to G2-nascent centrioles. As shown in Fig. 3, complete loss of CEP120 significantly reduced the targeting of Talpid3 (Fig. 3a) and C2CD3 (Fig. 3b) to the G2-nascent centrioles, but had no apparent effect on the localization of CP110 (Fig. 3c). The centriole pairs of KO-1 and KO-2 cells showed partially superimposed CP110 and SAS6 signals (yellow, Fig. 3c) with no loss of CP110 on their G2-nascent centrioles (Fig. 3c), indicating that CEP120-KO cells produce short centrioles but do not exhibit any alteration in the localization of CP110 to the distal ends of centrioles.

**Figure 4:**
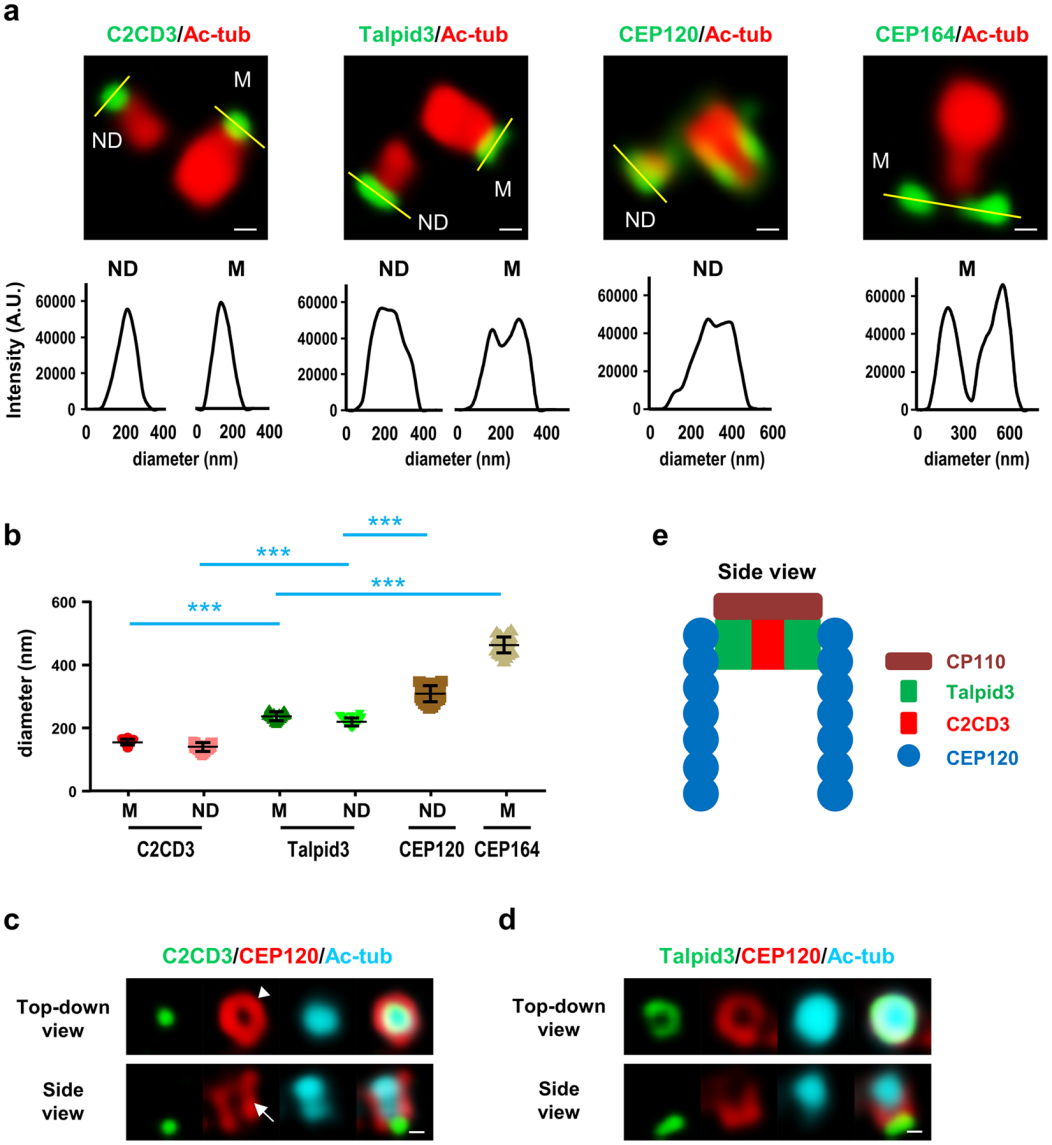
Super-resolution (3D-SIM) microscopic analysis of centriolar distal-end proteins. (**a)** RPE1 cells were synchronized at G2 phase and immunostained with the indicated antibodies. Lower panels show fluorescence profile plots. Representative images of more than ten cells are presented in (**a)**. ND: nascent daughter centriole, M: mother centriole. (**b)** The diameters of C2CD3, Talpid3, CEP120, and CEP164 protein spots (n = 30/each). Cumulative data from three independent experiments are shown. ***P < 0.001. (**c**,**d**) 3D-SIM analysis of the spatial localizations of C2CD3 (**c**), Talpid3 (**d**) and CEP120 (**c**,**d**) at the distal ends of centrioles. RPE1 cells were synchronized at G2 phase and immunostained with the indicated antibodies. Scale bar, 100 nm in (**a**,**c**,**d**). (**e**) Schematic illustration of the spatial localizations of C2CD3, Talpid3, CEP120, and CP110 at the distal ends of centrioles.

To precisely examine the spatial localization of these distal-end proteins in G2-nascent centrioles, we performed three-dimensional structured illumination microscopy (3D-SIM) with the indicated antibodies to examine their relative positions on mother (M) and nascent daughter (ND) centrioles in G2-phase cells (for representative images, see Fig. 4a). The diameters (nm) of the fluorescent spots derived from C2CD3, Talpid3, CEP120, and CEP164 staining in either M or ND centrioles were calculated and summarized in Fig. 4b. Since CEP120 is a daughter centriole-enriched protein^24^ and CEP164 is a mother centriole protein^36^, we measured the diameters of CEP120 and CEP164 signals on the ND and M centrioles, respectively (Fig. 4a). 3D-SIM analysis of ND centrioles showed that a portion of the CEP120 signal formed a ring-like structure that embraced the C2CD3 signal at the distal end of the G2-centriole (top-down view, Fig. 4c, arrow). Meanwhile, side-view images of the CEP120 signal often revealed a rather random elongated shape located outside the acetyl-tubulin-labeled centriole (side view, Fig. 4c, arrowhead). Interestingly, Talpid3 also formed a ring-like structure that partially overlapped with that formed by CEP120, but had a smaller diameter (top-down view, Fig. 4b,d). In contrast to CEP120, the signals of C2CD3 and Talpid3 were only detected at the distal ends of centrioles, and were absent from the centriole wall when viewed from the side (Fig. 4c,d, bottom). A recent report indicated that CP110 may be found at the distal ends of centrioles above C2CD3^47^. Taken together, the previous report and our present findings suggest that C2CD3 is located at the center of the centriole’s distal end, surrounded by Talpid3 followed by CEP120 (Fig. 4e). The centriole has an outmost layer formed by CEP164 (Fig. 4b), which is a known distal appendage marker for the mother centriole, and the distal ends of centrioles are capped by CP110. A similar finding was also noted in a very recent report^23^. Based on our present findings, we conclude that when cells lose CEP120, they experience impairments in the recruitment of C2CD3 and Talpid3, but not CP110, to the distal ends of centrioles.

### CEP120 interacts with C2CD3 and Talpid3, but not with OFD1

**Figure 5:**
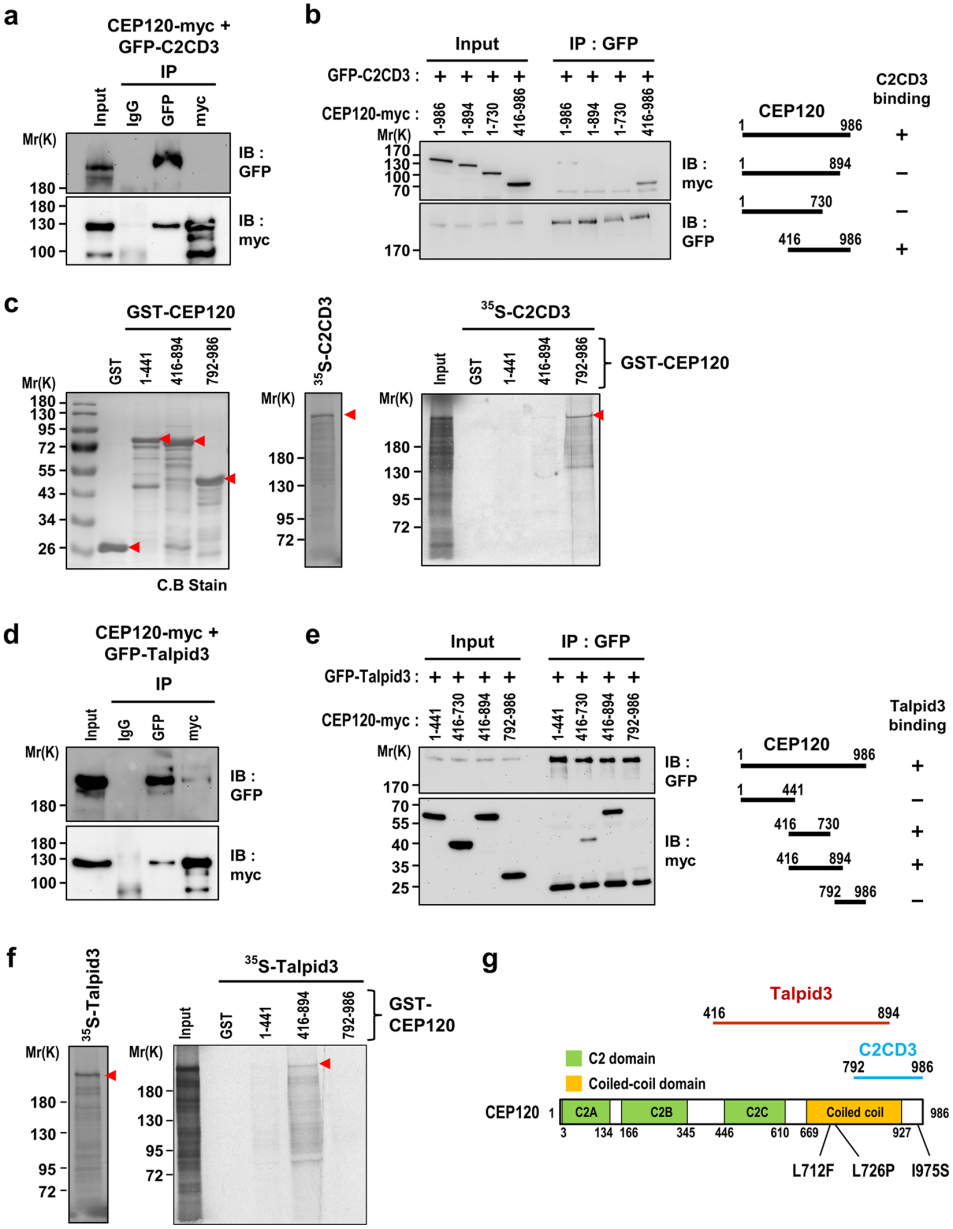
CEP120 interacts with C2CD3 and Talpid3. (**a)** CEP120 physically associates with C2CD3 *in vivo*. HEK293T cells were co-transfected with CEP120-myc and GFP-C2CD3 constructs. Twenty-four hours after transfection, cell lysates were immunoprecipitated (IP) and then immunoblotted (IB) with the indicated antibodies. (**b**) Mapping the C2CD3-interacting domain in CEP120. HEK293T cells were co-transfected with GFP-C2CD3 and various myc-tagged CEP120 constructs, analyzed by IP, and subsequent IB using the indicated antibodies. (**c**) CEP120 directly interacts with C2CD3. GST and various GST-CEP120 recombinant proteins were affinity purified (left). The full-length ^35^S-methionine-labeled C2CD3 proteins (middle) were incubated with bead-bound GST or various GST-CEP120 recombinant proteins (right) and analyzed by SDS-PAGE and autoradiography. (**d**) CEP120 physically associates with Talpid3 *in vivo*. HEK293T cells were co-transfected with CEP120-myc and GFP-Talpid3 constructs, and analyzed as described in (**a**). (**e**) Mapping the Talpid3-interacting domain in CEP120. HEK293T cells were co-transfected with GFP-Talpid3 and various myc-tagged CEP120 constructs and analyzed as described in (**b**). Uncropped blots of (**a**,**b**,**d**,**e**) are shown in Fig. S6b–e, respectively. (**f**) CEP120 directly interacts with Talpid3. The full-length ^35^S-methionine-labeled Talpid3 proteins (left) were incubated with bead-bound GST or various GST-CEP120 recombinant proteins (right) and analyzed by SDS-PAGE and autoradiography. (**g**) Schematic summary of the interactions of CEP120, C2CD3, and Talpid3, and the positions of the disease-associated CEP120 mutations that cause JS.

As our results showed that CEP120 is required to recruit C2CD3 and Talpid3 to the distal ends of centrioles (Fig. 3a,b), we next examined whether CEP120 could interact with C2CD3. Indeed, our co-immunoprecipitation (co-IP) results showed that full-length CEP120-myc could form a complex with GFP-C2CD3 (Fig. 5a). To further map the C2CD3-interacting region of CEP120, we co-transfected HEK 293 T cells with constructs encoding full-length GFP-C2CD3 plus a series of myc-tagged CEP120 truncation constructs, and performed co-IP experiments using an anti-GFP antibody. Our results showed that GFP-C2CD3 could co-precipitate with full-length CEP120-myc (aa 1–986) and CEP120-myc (aa 416–986), but not CEP120-myc (aa 1–894) or CEP120 (aa 1–730), implying that the C-terminal region of CEP120 (aa 416–986) could form a complex with C2CD3 *in vivo* (Fig. 5b). A direct interaction between CEP120 and C2CD3 was demonstrated by a GST pulldown assay, in which *in vitro*-translated ^35^S-methionine-labeled full-length C2CD3 was found to be directly and specifically pulled down by GST-CEP120 (aa 792–986) (Fig. 5c, right panel). In contrast, GST alone or the N-terminal region of GST-CEP120 (aa 1–441) failed to generate any detectable band (Fig. 5c), while GST-CEP120 (aa 416–894) generated a very weak band. These findings suggest that a direct C2CD3-interacting region is located at the C-terminus of CEP120 (aa 792–986).

Since previous co-IP results indicated physical associations of CEP120 with Talpid3^29^ and C2CD3 with OFD1^22^, we examined whether CEP120 could directly interact with Talpid3 or OFD1. Our co-IP results showed that full-length CEP120-myc could co-precipitate with GFP-Talpid3 (Fig. 5d) in transfected cells, which is consistent with the previous report^29^. We then further narrowed the Talpid3-associating domain to the middle regions of CEP120 (aa 416–730 and aa 416–894) (Fig. 5e). We used GST-CEP120 (aa 416–894) in a pulldown experiment and found that it could directly pulldown ^35^S-methionine-labeled full-length Talpid3 (Fig. 5f). Unexpectedly, when we included 0.5% sodium deoxycholate (SDC, an ionic detergent) in the reaction buffer, the ^35^S-methionine-labeled Talpid3 failed to bind the GST-CEP120 proteins (Fig. S4a), suggesting that the interaction between CEP120 (aa 416–894) and Talpid3 is sensitive to ionic detergent. In contrast, GST-CEP120 (aa 792–986) continued to interact with ^35^S-methionine-labeled C2CD3 in the presence of SDC (Fig. S4b). Furthermore, we detected no direct interaction between various GST-CEP120 recombinant proteins and ^35^S-methionine-labeled full-length OFD1 in reaction buffer without (Fig. S4c) or with (data not shown) 0.5% SDC. Together, our results indicate that CEP120 interacts with C2CD3 via its C-terminal region (aa 792–986) and Talpid3 through its middle region (aa 416–894), and CEP120 does not appear to interact with OFD1.

### Disease-associated mutations in CEP120 (L712F, L726P, and I975S) do not affect centriole elongation, but perturb cilia formation to various degrees

**Figure 6:**
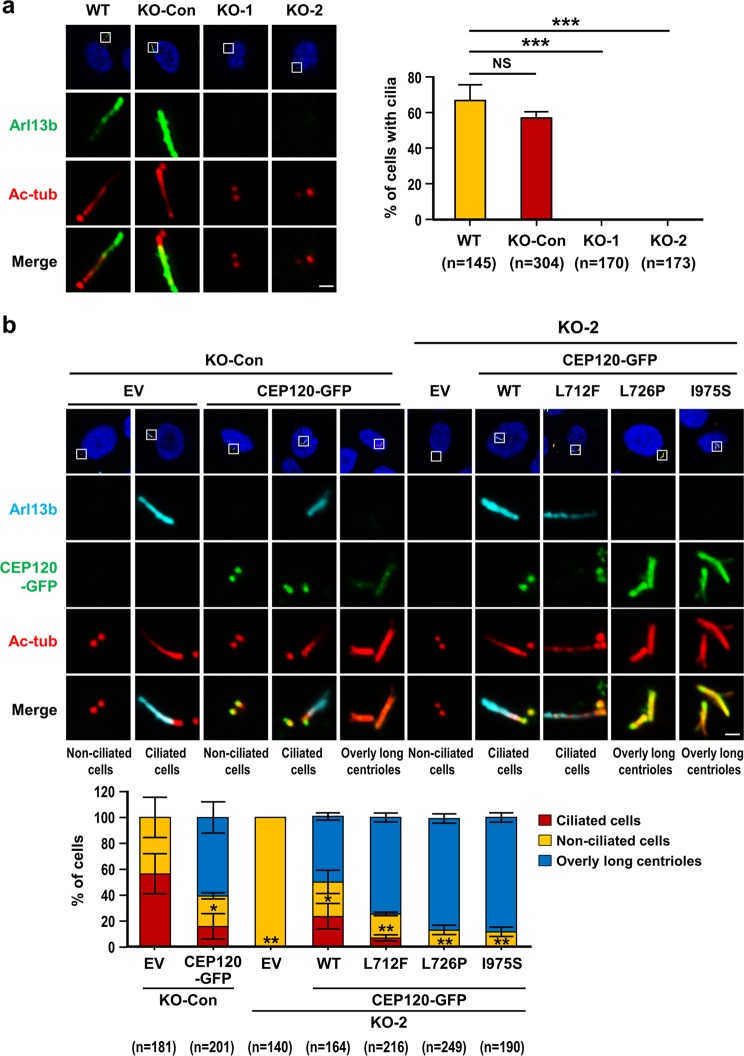
Various disease-associated CEP120 mutants impair cilia formation to different degrees. (**a**) Loss of CEP120 completely blocks cilia formation. RPE1-based WT (*p53*+/+*; CEP120*+/+), KO-Con (*p53−/−; CEP120*+/+), KO-1 (*p53−/−; CEP120−/−*), and KO-2 (*p53−/−; CEP120−/−*) cells were examined by immunofluorescence confocal microscopy using antibodies against Arl13b (a cilium marker, green) and acetyl-tubulin (Ac-tub, red). Histogram illustrating the percentages of cilia-containing cells. Error bars represent the mean ± s.d. from pools of cells (n) from three independent experiments. ***P < 0.001; NS, not significant. (**b**) Rescue experiments. KO-Con, KO-2, and KO-2 cells expressing doxycycline-inducible WT or mutant CEP120-GFP proteins were serum starved for 48 h to induce cilia formation. Ciliated cells (marked with long Arl13b/Ac-tub filaments), non-ciliated cells (Arl13b-negative), and cells with overly long centrioles (marked with long CEP120-GFP/Ac-tub filaments of >0.5 μm) were examined by confocal fluorescence microscopy using the indicated antibodies. Histogram illustrating the percentages of ciliated cells, non-ciliated cells, and cells with overly long centrioles. Error bars represent the mean ± s.d. from pools of cells (n) from three independent experiments. *P < 0.05; **P < 0.01. Note that CEP120-GFP overexpression induces overly long centrioles (>0.5 μm) that perturb cilia formation in the rescue experiments. EV: empty vector. Scale bar, 1 μm.

Recently, three CEP120 missense mutations (L712F, L726P, and I975S) located within the Talpid3 (aa 416–894)- and C2CD3 (aa 792–986)-interacting regions of CEP120 (Fig. 5g) were reported in human JS patients with complex clinical phenotypes^27^. We and others previously reported that overexpression of CEP120 or C2CD3 triggers centriole hyper-elongation^16,17,22^ and that C2CD3 is also required for cilia formation^48^. To examine whether the above-listed JS-associated mutations affect centriole elongation and cilia formation, we produced RPE1-based CEP120-GFP-inducible cell lines under doxycycline control in *CEP120−/−; p53−/−* background to induce the expression of GFP-tagged wild-type CEP120 or various CEP120 mutants. As shown in Supplementary Fig. 5, all three CEP120 mutants produced extra-long centrioles (>0.5 μm) similar to those found in CEP120 wild-type overexpressing cells, suggesting that these mutations do not affect centriole elongation activity. Further analysis showed that the formation of cilia was completely blocked in KO-1 and KO-2 cells (*CEP120−/−; p53−/−*; Fig. 6a), but not in KO-Con cells (*p53−/−*; Fig. 6a), suggesting that CEP120 is required for cilia formation and the *p53*-null mutation used herein does not affect ciliogenesis. Unexpectedly, the cilia-null phenotype was only partially rescued by exogenous expression of wild-type *CEP120-GFP* in KO-2 cells (KO-2 + CEP120-GFP; Fig. 6b). This low efficiency of cilia rescue could reflect that ciliogenesis is perturbed by the extra-long centrioles induced by excess CEP120. Indeed, a similar perturbation of cilia formation was frequently observed in KO-Con cells overexpressing CEP120-GFP (KO-Con + CEP120-GFP; Fig. 6b).

We further examined whether the JS-associated mutations affected cilia formation in the various CEP120-GFP-inducible cell lines. Our results showed that the cilia-null phenotype could be partially restored by exogenous expression of wild-type CEP120 or the L712F mutant, but not the L726P or I975S mutants (Fig. 6b). Together, our findings suggest that the naturally occurring JS-associated CEP120 mutants, L726P and I975S, impair cilia formation.

### JS-associated mutation I975S exhibits reduced binding to C2CD3 and impedes recruitment of C2CD3 to the centrioles

**Figure 7:**
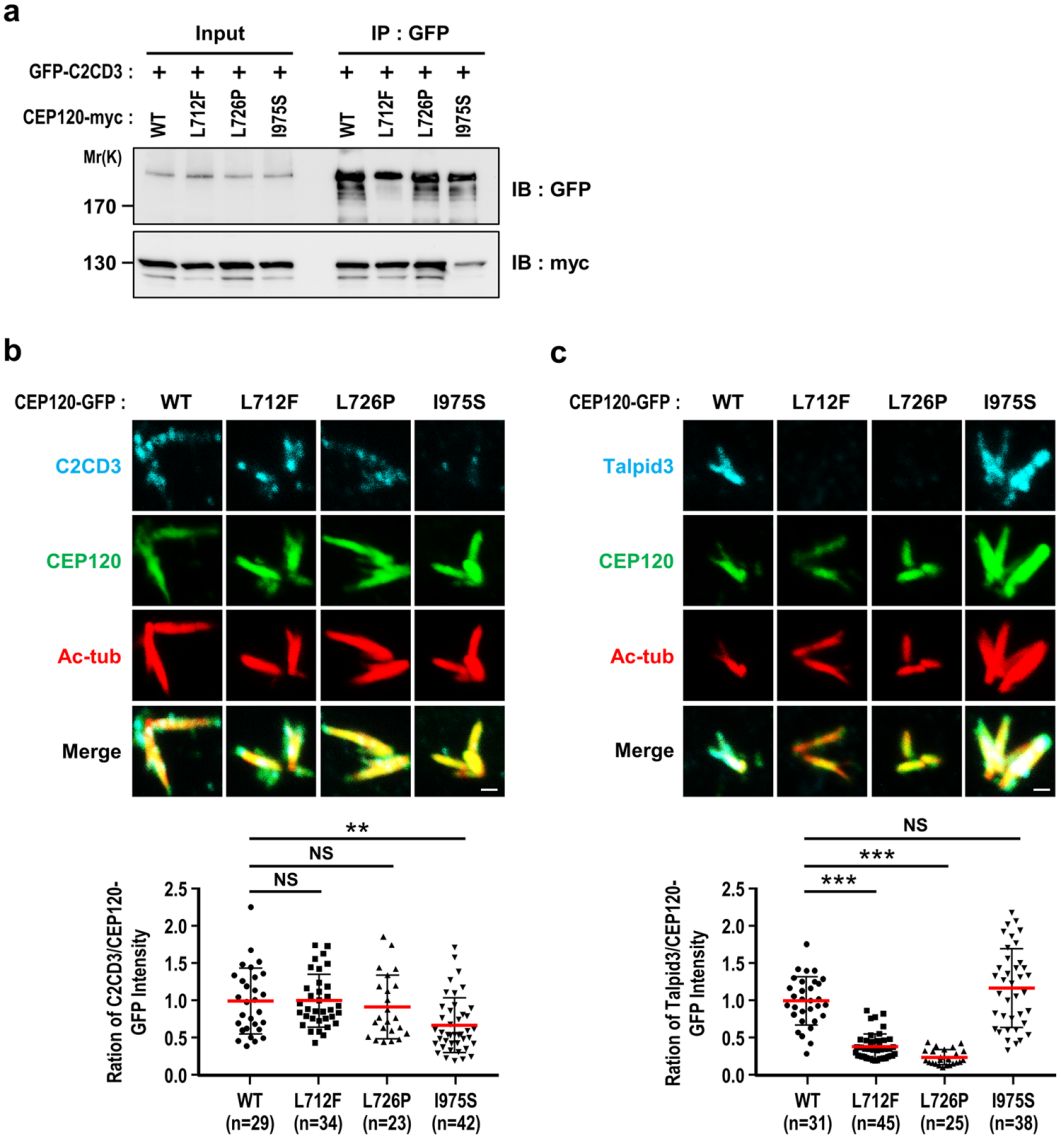
Disease-associated CEP120 mutants exhibit reduced binding to C2CD3 and impaired recruitments of C2CD3 and Talpid3 to the centrioles. (**a**) The naturally occurring I975S mutation of CEP120 exhibits reduced binding to C2CD3. HEK293T cells were co-transfected with GFP-C2CD3 and various myc-tagged CEP120 constructs (WT and mutants), analyzed by IP, and subsequent IB using the indicated antibodies. Uncropped blots are shown in Fig. S6f. (**b**,**c**) Disease-associated CEP120 mutations impair the recruitment of C2CD3 and Talpid3 to the centrioles. KO-2 cells expressing doxycycline-inducible CEP120-GFP (WT or L712F, L726P, and I975S mutants) were synchronized at G2, fixed, and stained with the indicated antibodies. Since C2CD3 and Talpid3 interact with CEP120 and co-distribute with the CEP120-induced overly long centrioles, the intensities of C2CD3 and Talpid3 were normalized to the intensity of CEP120-GFP. Histogram illustrating the intensity ratios of C2CD3 to CEP120-GFP (**b**) and Talpid3 to CEP120-GFP (**c**). Error bars represent the mean ± s.d. from pools of cells (n) from three independent experiments. **P < 0.01; ***P < 0.001; NS, not significant. Scale bar, 1 μm.

Our present results show that the JS-associated mutations, L712F, L726P, and I975S, are located within the Talpid3 (aa 416–894)- and C2CD3 (aa 792–986)-interacting regions of CEP120 (Fig. 5g), and the L726P and I975S mutants have an interference effect on ciliogenesis (Fig. 6b). To investigate whether these JS-associated mutations exhibit decreased binding to Talpid3 and/or C2CD3, we co-transfected HEK293T cells with GFP-C2CD3 and various CEP120-myc constructs (WT or L712F, L726P, or I975S mutants), and the cell lysates were analyzed by co-IP assays. As shown in Fig. 7a, the I975S mutant had a reduced binding affinity for C2CD3, whereas the L712F and L726P mutants did not show any obvious alteration in C2CD3 binding (Fig. 7a). Consistent with this finding, a GST pulldown assay demonstrated that there was a dramatic reduction of the interaction between ^35^S-methionine-labeled C2CD3 and the I975S mutant under the more stringent condition (0.5% SDC) (Fig. S4d). Surprisingly, our GST pulldown assay revealed no apparent difference in the binding of ^35^S-methionine-labeled Talpid3 to wild-type or mutant CEP120 in the presence (Fig. S4e) or absence (Fig. S4f) of 0.5% SDC. Taken together, these results suggest that the JS-associated mutant, I975S, exhibits a specific reduction in C2CD3 binding, while the other two mutants (L712F, L726P) have no apparent alteration in their binding of C2CD3 or Talpid3.

Although, the intensities of Talpid3 (Fig. 3a) and C2CD3 (Fig. 3b) at the distal ends of centrioles were significantly reduced in KO-1 and KO-2 cells, we found there have been no significant changes of their corresponding protein levels between KO-Con and KO-2 cells (Fig. 3d). This suggests that the loss of CEP120 affects the centriolar localization but not the protein stability of Talpid3 and C2CD3. Accordingly, we examined the centriolar localization of Talpid3 and C2CD3 in various CEP120 mutant-inducible cell lines. Since CEP120 overexpression induces centriole over-elongation, the relative intensities of Talpid3 or C2CD3 on the centrioles were normalized to the intensity of CEP120. Our results showed that the I975S mutant triggered a significant decrease in the centriolar localization of C2CD3 (Fig. 7b). Unexpectedly, the L712F and L726P mutants, but not the WT protein or the I975S mutant, reduced the centriolar localization of Talpid3 (Fig. 7c). Collectively, our results support the idea that the JS-associated mutant, I975S, exhibits reduced binding to C2CD3 and impedes recruitment of C2CD3 to the centrioles, whereas the L712F and L726P mutants show no apparent alteration in their bindings of Talpid3, but significantly inhibit Talpid3 targeting to centrioles.

## Discussion

**Figure 8:**
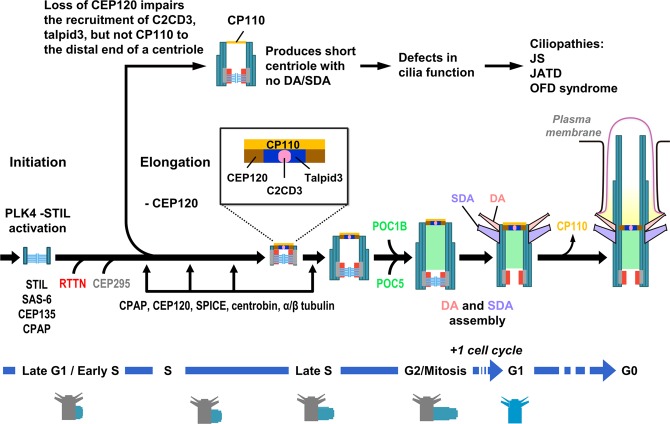
Model showing the role of CEP120 in procentriole elongation, centriole appendage assembly, and ciliogenesis. For details, see the Discussion.

We are just starting to understand the molecular mechanism through which an immature centriole acquires DAs and SDAs to become a mature mother centriole. Our group and others previously reported that CEP120 is a daughter centriole-enriched protein that plays an essential role in centriole elongation^16,17^. Here, we uncover a new function of CEP120 by showing that it recruits C2CD3 and Talpid3 to the distal end of a newborn centriole, in a step that is essential for later DA and SDA assembly and ciliogenesis. We also found that the JS-associated CEP120 mutant, I975S, shows a low affinity for C2CD3 binding and triggers impairments in cilia formation. Importantly, our findings delineate the function of the CEP120-C2CD3-Talpid3 axis in the assembly of DAs and SDAs during centriole biogenesis and provide a causal relationship to explain why mutations in three different centriolar protein-encoding genes (*CEP120, C2CD3*, and *Talpid3*) cause clinically related phenotypes (see below). Based on our current results and previous publications, we propose a possible model for the interplay of CEP120 with C2CD3 and Talpid3 in centriole biogenesis (Fig. 8).

In this model (Fig. 8), centriole assembly occurs during late G1/early S phase after PLK4-STIL activation^9–14^, followed by the assembly of the SAS6-CEP135 containing cartwheel and the recruitment of CPAP and RTTN to the proximal end of pre-existing centrioles *via* the STIL-CPAP^49^ and STIL-RTTN^32^ interactions. RTTN putatively stabilizes a primitive procentriole body (PPB) containing STIL, CPAP, and SAS-6, which is essential for CPAP-mediated centriole elongation and the CEP295-dependent loading of POC5/POC1B to the distal half of the centriole at later stages^32^. During early S-G2 phase, CPAP cooperates with CEP120 to promote procentriole elongation^16,17^, while the latter is required to recruit other centriole elongation factors, such as SPICE (Fig. 2c) and centrobin (Fig. 2d), to the procentrioles. The loss of CEP120 has no apparent effect on the targeting of the centriolar inner lumen protein, RTTN (Fig. 2b), or CEP295 (Fig. 2a) to the proximal ends of newborn centrioles. Importantly, we found that CEP120 is critical for the initial recruitment of C2CD3 and Talpid3, but not CP110, to the distal ends of centrioles (Fig. 3), since complete loss of CEP120 significantly blocked the centriolar localizations of C2CD3 (Fig. 3b) and Talpid3 (Fig. 3a). Thus, the loss of CEP120 impairs the recruitment of centriole elongation factors (SPICE and centrobin), later-born centriolar proteins (POC1B and POC5), and centriolar distal-end proteins (Talpid3 and C2CD3) to the newborn centrioles, resulting in the production of short centrioles with defective DAs and SDAs. The structurally defective centrioles without functional DAs could impair the initial step of ciliogenesis by interfering the docking of myosin-Va-associated preciliary vesicles to the centriole^6^. Furthermore, the complete loss of CEP120 had no apparent effect in removing CP110, a negative regulator of ciliogenesis^43^, from the distal end of mother and daughter centrioles (Fig. 3c) during ciliogenesis thereby further preventing cilia formation.

Numerous *CEP120* gene mutations have recently been identified in human patients, including those with JATD, JS, and complex ciliopathy and neurological phenotypes^26,27^. JATD is a skeletal dysplasia characterized by a range of skeletal and extra-skeletal anomalies, while JS manifests a mainly neurological phenotype consisting of hypotonia, cognitive impairment, and severe hypoplasia of the cerebellar vermis^26,27^. Here, we examined three disease-associated mutations (L712F, L726P, and I975S) located within the Talpid3 (aa 416–894)- and C2CD3 (aa 792–986)-interacting regions of CEP120 (Fig. 5g). We found that the three mutants had no obvious effect on centriole elongation (Fig. S5). Interestingly, the I975S mutation, which is located in the C2CD3-interacting domain of CEP120, reduced the binding of CEP120 to C2CD3 (Figs 7a; S4d), impaired the recruitment of C2CD3 to the centrioles (Fig. 7b), and was associated with defective cilia formation (Fig. 6b). Although, the L712F and L726P mutations are located within the Talpid3-interacting domain, they did not appear to interfere with the binding of CEP120 to Talpid3 (Fig. S4f). Instead, they significantly inhibited the recruitment of Talpid3 to the centrioles (Fig. 7c). The reason for this is not yet clear. It is possible that these two mutations do not directly alter the CEP120-Talpid3 interaction, but rather induce a conformational change of CEP120 that interferes with the recruitment of Talpid3 to the centrioles. Alternatively, CEP120 may interact with other yet-unidentified proteins that are essential for the proper localization of Talpid3 and these interactions are altered by the L712F and L726P mutations. Future identification of new CEP120-binding partners and X-ray crystallography of the CEP120 protein complexes may resolve this question. Previous publications indicated that overexpression of C2CD3 can induce centriole hyper-elongation and that loss of C2CD3 produces short centrioles without SDAs and DAs^22^, in a pattern similar to that observed in CEP120-overexpressing^17^ or CEP120-knockout cells (this report). Interestingly, a very recent report proposed that the C2CD3-Talpid3 complex may serve as a multi-functional hub that is involved in centriole maturation and DA and SDA assembly^23^. Here, we show that CEP120 may directly interact and form a complex with C2CD3 (Fig. 5c) and Talpid3, (Fig. 5f), suggesting that CEP120 could mediate DA and SDA assembly through its interactions with C2CD3 and Talpid3.

Finally, recent reports showed that mutations in the *C2CD3* gene cause skeletal dysplasia, polydactyly, and ciliopathies^50,51^, while *Talpid3* mutations cause Joubert syndromes and Short-Rib polydactyly syndromes^52,53^. We herein showed that CEP120 directly interacts with C2CD3 (Fig. 5c) and Talpid3 (Fig. 5f), making it particularly notable that mutations in all three encoding genes reportedly give rise to a similar spectrum of ciliopathies, including the overlapping clinical features of JS and JATD^26,27,50–53^. Taken together, our findings illuminate the mechanism through which the CEP120-C2CD3-Talpid3 axis contributes to DA and SDA assembly during centriole biogenesis and delineate a causal relationship to explain why mutations in *CEP120*, *C2CD3*, and *Talpid3* cause clinically related phenotypes.

## Methods

### Plasmids

The GFP- or myc-tagged cDNA constructs for CEP120, including those encoding the full-length protein and various CEP120 fragments were as described in our previous paper^17^. The GST-fusion constructs were generated by inserting cDNAs encoding various potions of CEP120 in-frame with GST in the pGEX4T vector (GE Healthcare) or were as previously described^17^. The QuikChange site-directed mutagenesis kit (Stratagene) was used to make the disease-associated CEP120 mutant constructs. The constructs that express various GFP-CEP120 mutant proteins were generated using pLVX-Tight-puro vectors (BD Biosciences Clontech). The human C2CD3 cDNA fragment, which was a gift from Dr. Gonczy’s lab^54^, was subcloned into the pEGFP-C1 vector (BD Biosciences Clontech). The cDNAs encoding full-length Talpid3 or C2CD3 were obtained by RT-PCR from the total RNA of human HEK293T cells, and subcloned in-frame into the pEGFP-C1 vector (BD Biosciences Clontech). The sequences of all constructed plasmids were confirmed.

### Antibodies

The rabbit polyclonal antibody against CEP120 (residues 639–986) was as described in our previous paper^17^. The Alexa Fluor-conjugated CEP120 antibody was generated using an Alexa Fluor™ 568 Antibody Labeling Kit (A20184; Invitrogen). The antibody against Talpid3 was raised in rabbit using recombinant GST-Talpid3 (residues 1–149) and affinity purified. This study also used antibodies against the following: antibodies against CPAP (residues 1070–1338, 1:1000 dilution)^55^, RTTN (residues 1347–1591, 1:400 dilution)^32^, CEP295 (residues 2092–2430, 1:500 dilution)^40^, centrin 2 (residues 1–173, 1:1000 dilution)^46^, and centrobin (residues 443–626, 1:1000 dilution)^46^; hSAS-6 (H00163786; 1:100 dilution, mouse polyclonal Ab), CEP162 (PAB22408; 1:200 dilution)(all from Abnova); CP110 (12780-1-1p; 1:600 dilution), ARL13B (17711-1-AP; 1:1000 dilution)(all from Proteintech); hPOC5 (A303–341;1:500 dilution), CEP128 (A303-348A; 1:800 dilution), SPICE (A303–272;1:800 dilution) (all from Bethyl); CEP164 (NBP1-81445; 1:500 dilution), CEP83 (NBP1-90690; 1:200 dilution)(all from Novus Biologicals); acetylated tubulin (T6793; 1:500 dilution), STIL (HPA046543; 1:100 dilution), C2CD3 (HPA038552; 1:200 dilution), alpha-tubulin (T9026; 1:10000 dilution) (all from Sigma-Aldrich); hPOC1B (PA5-24495; 1:500 dilution; Thermo Fisher); GFP (632381; 1:3000 dilution; BD Bioscience); ODF2 (ab43840; 1:200 dilution; abcam); and Myc (4A6; 1:3000 dilution; EMD Millipore).

### Cell culture, transfection, and synchronization

We maintained HEK293T and U2OS cells (originally from ATCC) in Dulbecco’s modified Eagle’s (DME) medium containing 10% fetal bovine serum (FBS). Human telomerase-immortalized retinal pigment epithelial cells (RPE1) and p53-null RPE1^32^ were grown in DME/F12 (1:1) medium containing 10% FBS. Cell transfection was performed with Lipofectamine 2000 (Invitrogen) as described^32^. In synchronization study, we arrested cells at early S phase by addition of 2 μg/ml aphidicolin into the medium for 24 h. The cells were then released in fresh medium for another 15 h to enrich for G2-phase cells as previously described^32^. For induction of cilia formation, cells were arrested at G0 phase by incubation for 48 h in serum-free medium. All cell lines were examined and found no mycoplasma contamination.

### Production of CEP120-null cells using the CRISPR/Cas9 system

Since CEP120-null cells cannot survive in the presence of wild-type p53, we used CRISPR/Cas9-mediated gene targeting system^30^ to inactivate *CEP120* gene in RPE1-based p53-null cells as previously described^32^. The targeting sequences of *CEP120* gRNAs used for the CRISPR/Cas9 system were 5′-GTCGTCGTGTCCATCCTAGA-3′ and 5′-GTTTGCTACTGAGTTAGCTT-3′. We constructed the gRNA expression plasmids by inserting annealed primers into the gRNA cloning vector (plasmid #41824; Addgene). The p53-null RPE1 cells were nucleofected with 2.5 μg hCas9 plasmid (plasmid #41815; Addgene) and 2.5 μg gRNA according to the manufacturer’s instructions. Single colonies of nucleofected cells were picked and expanded by serially dilution as described^32^. Confocal immunofluorescence microscopy, Western blotting, and DNA sequencing were used to analyze the loss of CEP120 in CEP120-null cells.

### Generation of U2OS- or RPE1-based doxycycline-inducible cell lines

The U2OS-based doxycycline inducible PLK4-myc cell line used in this study was as previously described^40^. To obtain CEP120-null RPE1 cell lines inducibly expressing CEP120-GFP (WT or mutants), lentiviruses containing CEP120-GFP (WT or mutants) in the pLVX-tight-puro vector (BD Biosciences Clontech) were used to infect CEP120-null RPE1 Tet-On cells that stably express rtTA. The infected cells were selected with 10 μg/ml puromycin or were sterile-sorted by cell sorter (FACSAria, BD Biosciences) for GFP signal. The positive cells were selected and expanded as inducible lines. The expression of CEP120-GFP (WT or mutants) was induced by adding 1 μg/ml doxycycline to the culture medium.

### siRNA analysis

The siRNAs and the non-targeting siRNA control for CEP120 were obtained from Invitrogen (siCEP120#1 sequence 5-AAAUCAAAUGCACAGUAAGACUGGG-3)^17^. siRNA transfections were performed using Lipofectamine RNAiMAX (Invitrogen) according to the manufacturer’s protocol.

### GST pulldown assay

To examine direct protein-protein interactions, various GST-CEP120 truncated recombinant proteins were affinity purified by glutathione-agarose beads (Sigma-Aldrich) and a GST pulldown assay was performed as previously described^17^. Briefly, ^35^S-methionine labeled full-length C2CD3, Talpid3, or OFD1 were generated by *in vitro* transcription-translation using a TNT T7 Quick Coupled Transcription/Translation System (Promega) and incubated with immobilized GST-CEP120 recombinant proteins in EBC buffer (50 mM Tris–HCl, pH 8.0, 150 mM NaCl, 1% NP-40, 20 mM glycerophosphate, 0.3 mM Na_3_VO_4_, 1 μg/ml aprotinin, 1 μg/ml leupeptin, and 1 μg/ml pepstatin) with or without 0.5% sodium deoxycholate (SDC). GST was used as a negative control. The samples were washed, separated by SDS-PAGE, and analyzed by autoradiography.

### Microscopy

For immunofluorescence confocal microscopy, three-dimensional structured illumination microscopy (3D-SIM), and electron microscopy, cells were processed as previously described^32^. Briefly, cells on coverslips were treated with aphidicolin for synchronization at the G1/S phase transition and released with fresh medium at the indicated times. The cells were fixed in methanol and incubated with the indicated primary antibodies. After wash, the cells were incubated with Alexa Fluor 488-, Alexa Fluor 568-, or Alexa Fluor 647-conjugated secondary antibodies (Invitrogen). In some experiments, cells were immunostained with the Alexa Fluor 568-conjugated CEP120 antibody. The samples were viewed on a confocal system (LSM 700 or LSM880 Airyscan systems; Carl Zeiss). The 3D-SIM super-resolution images were acquired using a Zeiss ELYRA system equipped with a Plan Apochromat 63 × /1.4NA oil-immersion objective and the ZEN software (Carl Zeiss). For electron microscopy (EM), cells were grown on Aclar film (Electron Microscopy Sciences), fixed, and embedded in Spurr’s resin as previously described^32^. Thin sections (100 nm) were stained with 4% uranyl acetate and Reynold’s lead citrate and the samples were examined with an electron microscope (T FEG-TEM; FEI Tecnai G2 TF20 Super TWIN)^32^.

### Statistical analysis

Statistical analyses were performed using GraphPad Prism 6 and results are presented as mean ± standard derivation (s.d.). Statistical differences between two data sets were analyzed using the two-tailed unpaired Student’s t-test. The P values (*P < 0.05, **P < 0.01, and ***P < 0.001) are considered to be statistically significant. NS, not significant.

## Supplementary information

Supplementary Infomation

## Data Availability

All data supporting the findings of this study can be found within the paper and its Supplementary Information files.
