# Author Correction: CEP120 interacts with C2CD3 and Talpid3 and is required for centriole appendage assembly and ciliogenesis

**DOI:** 10.1038/s41598-020-58151-y

**Published:** 2020-01-22

**Authors:** Jhih-Jie Tsai, Wen-Bin Hsu, Jia-Hua Liu, Ching-Wen Chang, Tang K. Tang

**Affiliations:** Institute of Biomedical Sciences, Academia Sinica, Taipei, Taiwan

Correction to: *Scientific Reports* 10.1038/s41598-019-42577-0, published online 15 April 2019

This Article contains an error in Fig. 2a (KO-2). The panels for KO-2/CEP295 are in the wrong order (up/down images are misplaced). The correct Fig. 2a for KO-2 appears below as Fig. 1.

**Figure 1:**
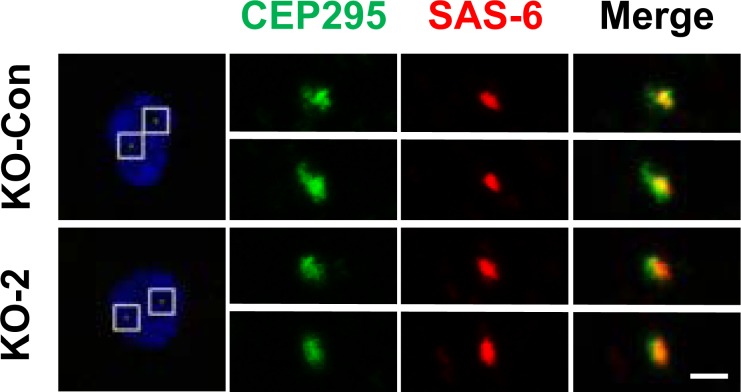
.

