# Supplementary material for: CEP120 interacts with C2CD3 and Talpid3 and is required for centriole appendage assembly and ciliogenesis
Source: Sci Rep. 2019 Apr 15;9:6037. doi: 10.1038/s41598-019-42577-0 (PMC6465297; doi:10.1038/s41598-019-42577-0)
Supplement: Supplementary file 1 — Supplementary Infomation [file 41598_2019_42577_MOESM1_ESM.pdf]

Supplementary Information

CEP120 interacts with C2CD3 and Talpid3 and is required for centriole appendage assembly and ciliogenesis

Jhih-Jie Tsai, Wen-Bin Hsu, Jia-Hua Liu, Ching-Wen Chang, and Tang K Tang*

Institute of Biomedical Sciences, Academia Sinica, Taipei, Taiwan

*Corresponding author

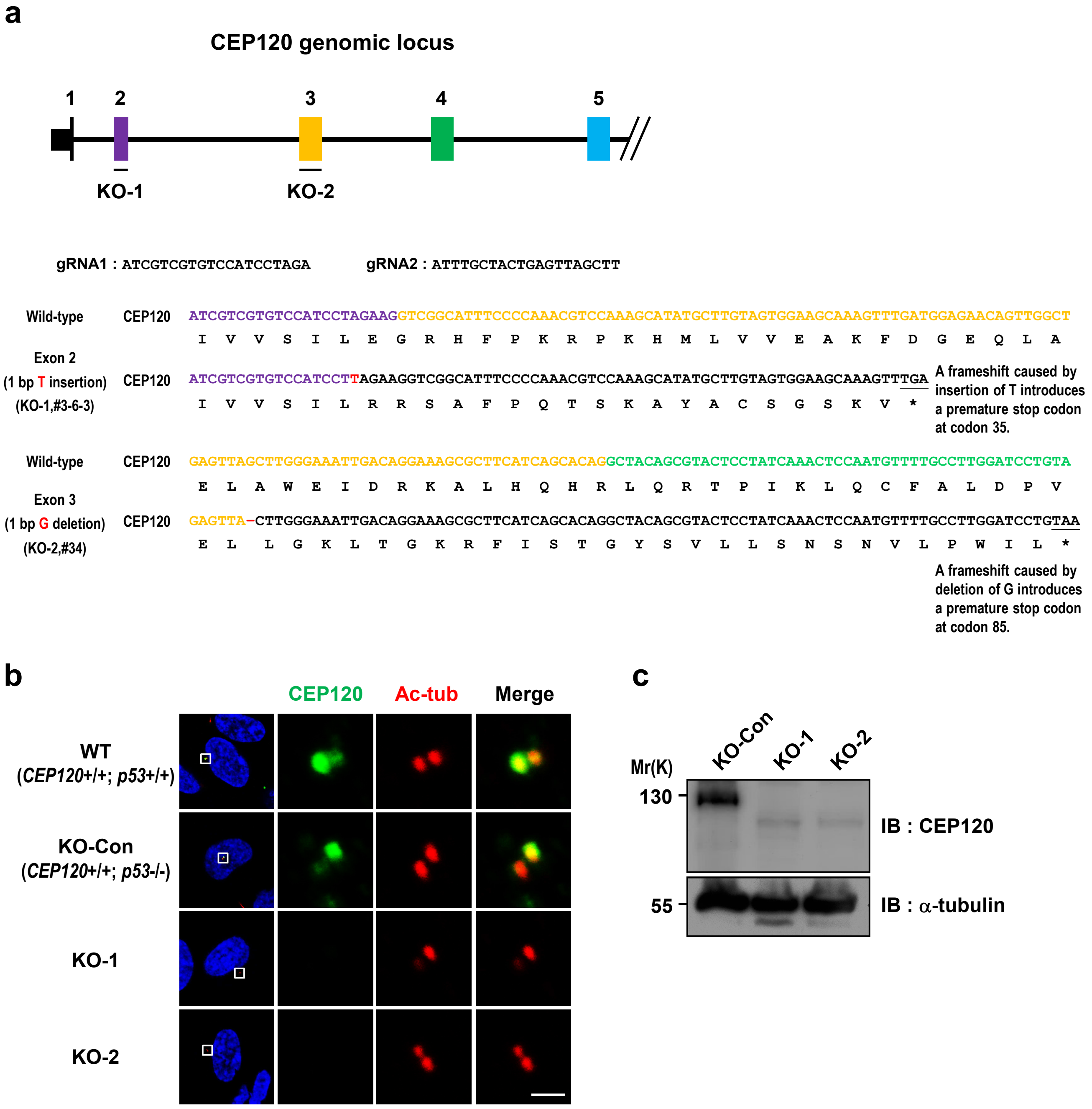

Supplementary Fig. S1. Generation and analysis of *CEP120* knockout cells. **a** Diagram of exons 1-5 of the 20 exons in the human *CEP120* genomic locus, showing the location of the guide RNAs (gRNAs) used for CRISPR-directed genome editing (KO-1 and KO-2; not to scale). Exons are indicated in color to clarify splicing. (top sequences) Genome editing of the *CEP120* genomic sequence, and the resultant transcript and predicted protein sequences for KO-1 (exon 2 gRNA). Sequences are colored according to their respective exon, and part of the WT exon 2 sequence is omitted. The lesion (deletion or insertion) is shown in red. (bottom sequences) Genome editing of the *CEP120* genomic sequence, and the resultant transcript and predicted protein sequences for KO-2 (exon 3 gRNA). The lesion (deletion or insertion) is shown in red. **b** RPE1-based WT (*CEP120+/+; p53+/+*), KO-Con (*CEP120+/+; p53-/-*), KO-1 (*CEP120-/-; p53-/-*), and KO-2 (*CEP120-/-*; *p53-/-*) cells were immunostained with antibodies against CEP120 (green) and Ac-tubulin (red). **c** KO-Con, KO-1, and KO-2 cells were subjected to Western blot analysis with the indicated antibodies. Uncropped blots are shown in Fig. S6g. Scale bar, 1 μm.

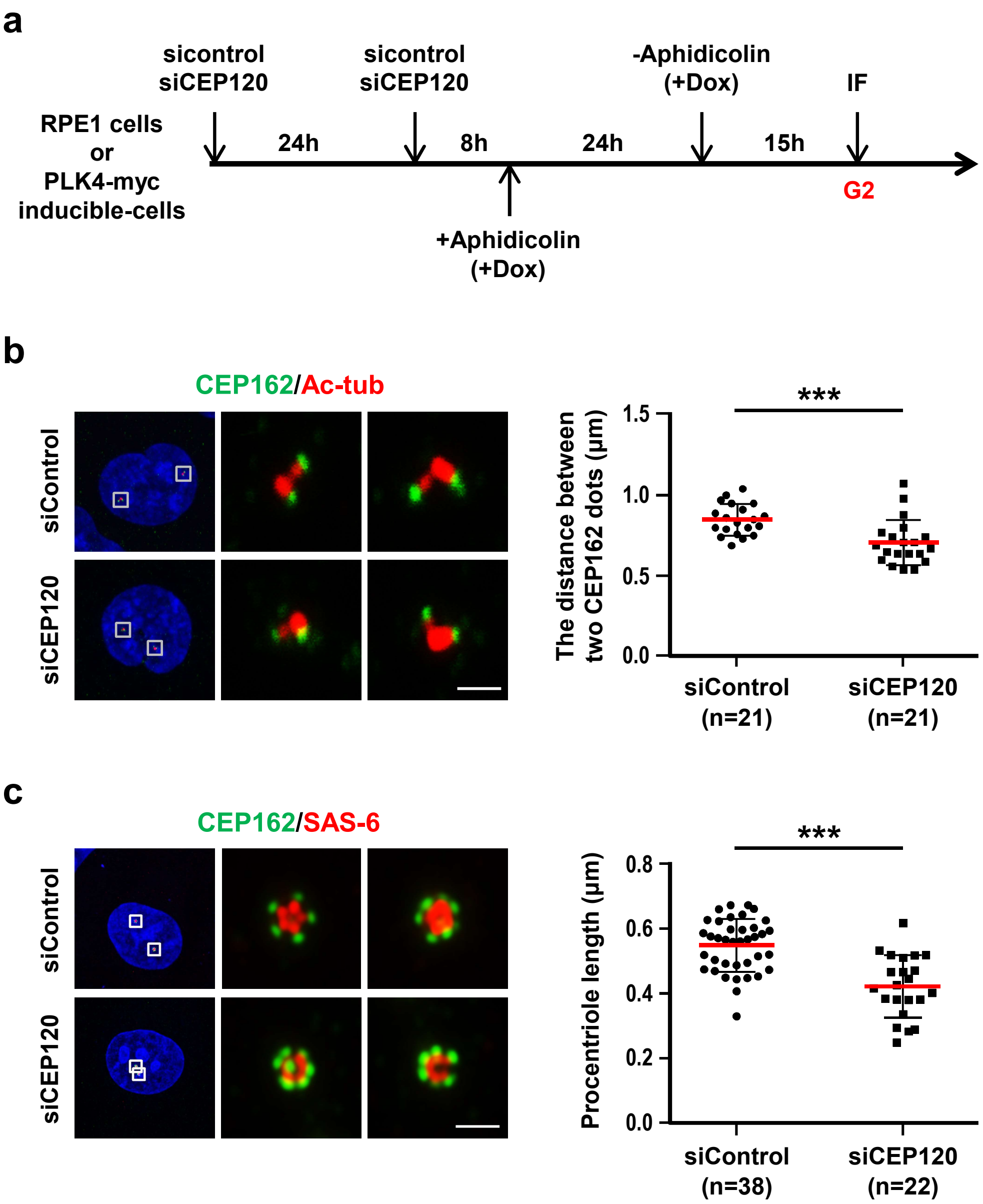

Supplementary Fig. S2 Depletion of CEP120 by siRNA treatment perturbs centriole elongation. **a-c** RPE1 (**b**) or PLK4-myc-doxycycline (Dox)-inducible cells (**c**) were treated with siControl or siCEP120 as shown in **a**, and analyzed by confocal fluorescence microscopy using the indicated antibodies. The distances between the CEP162-positive dots associated with a given pair of orthogonally oriented centrioles in siCEP120-treated RPE1 cells were calculated and are shown in **b**. The procentriole length in **c** was measured as the distance between the fluorescent peak intensity of SAS-6 (red) and CEP162 (green) in siControl and siCEP120-treated cells. Error bars represent the mean ± s.d from pools of cells (n) from three independent experiments. ***P < 0.001 (two-tailed unpaired t-test). Scale bar, 1 μm.

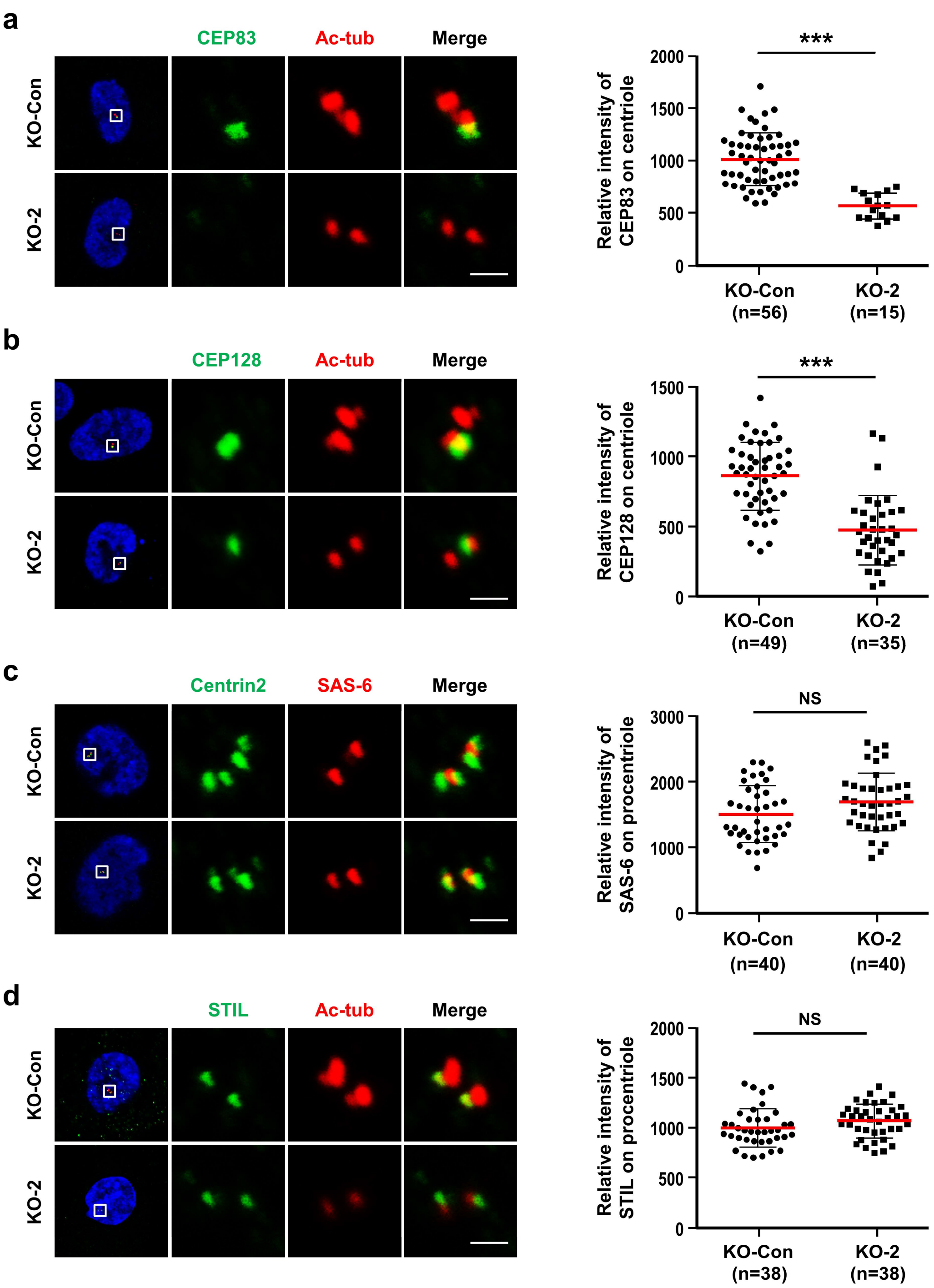

Supplementary Fig. S3. CEP120 loss perturbs the recruitments of CEP83 (**a**) and CEP128 (**b**) to the centriole appendages, but does not alter the localizations of the early-born centriolar proteins, SAS-6 (**c**) and STIL (**d**), to the procentrioles. KO-Con and KO-2 cells were synchronized at early S phase and analyzed by confocal fluorescence microscopy using the indicated antibodies, and the results were quantified. Histogram illustrating the relative intensities of CEP83 (**a**), CEP128 (**b**), SAS-6 (**c**), and STIL (**d**) on centrioles. Error bars represent the mean $\pm$ s.d. from pools of cells (n) from three independent experiments. ***P < 0.001; NS, not significant (two-tailed unpaired t-test). Scale bar, 1 μm.

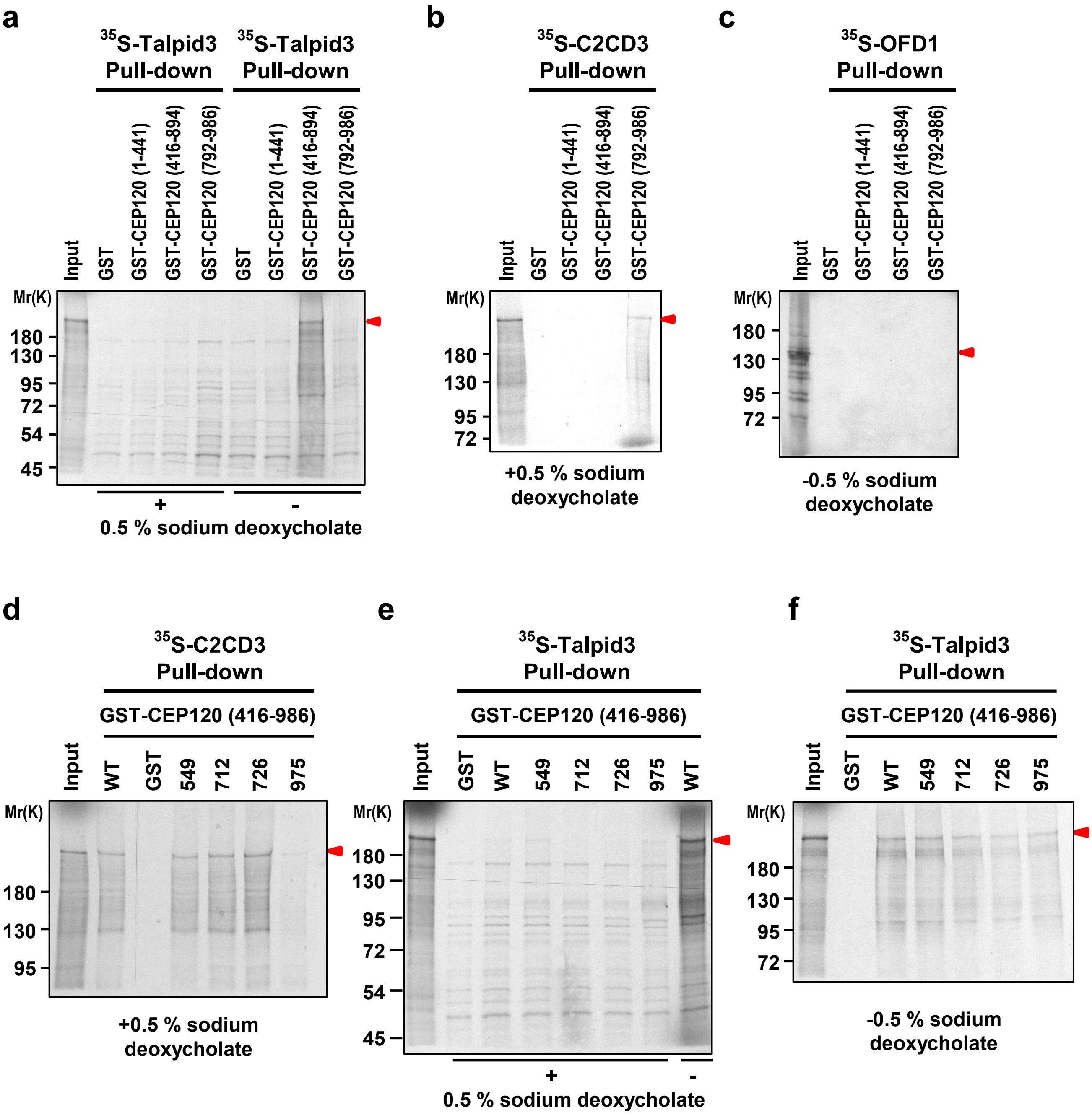

Supplementary Fig. S4. CEP120 interacts with C2CD3 and Talpid3, and does not detectably interact with OFD1. **a-c** Full-length ^{35}S -methionine-labeled Talpid3 (**a**), C2CD3 (**b**), or OFD1 (**c**) proteins were incubated with bead-bound GST or various GST-CEP120 recombinant proteins with (**a, b**) or without (**a, c**) 0.5% sodium deoxycholate (SDC). The samples were washed and analyzed by SDS-PAGE and autoradiography. **d-f** The JS-associated CEP120 mutant (I975S) exhibits reduced binding to C2CD3 but not Talpid3. Full-length ^{35}S -methionine-labeled C2CD3 (**d**) or Talpid3 (**e, f**) proteins were incubated with bead-bound GST or various GST-CEP120 recombinant proteins with (**d, e**) or without (**e, f**) 0.5% sodium deoxycholate, and analyzed as described above.

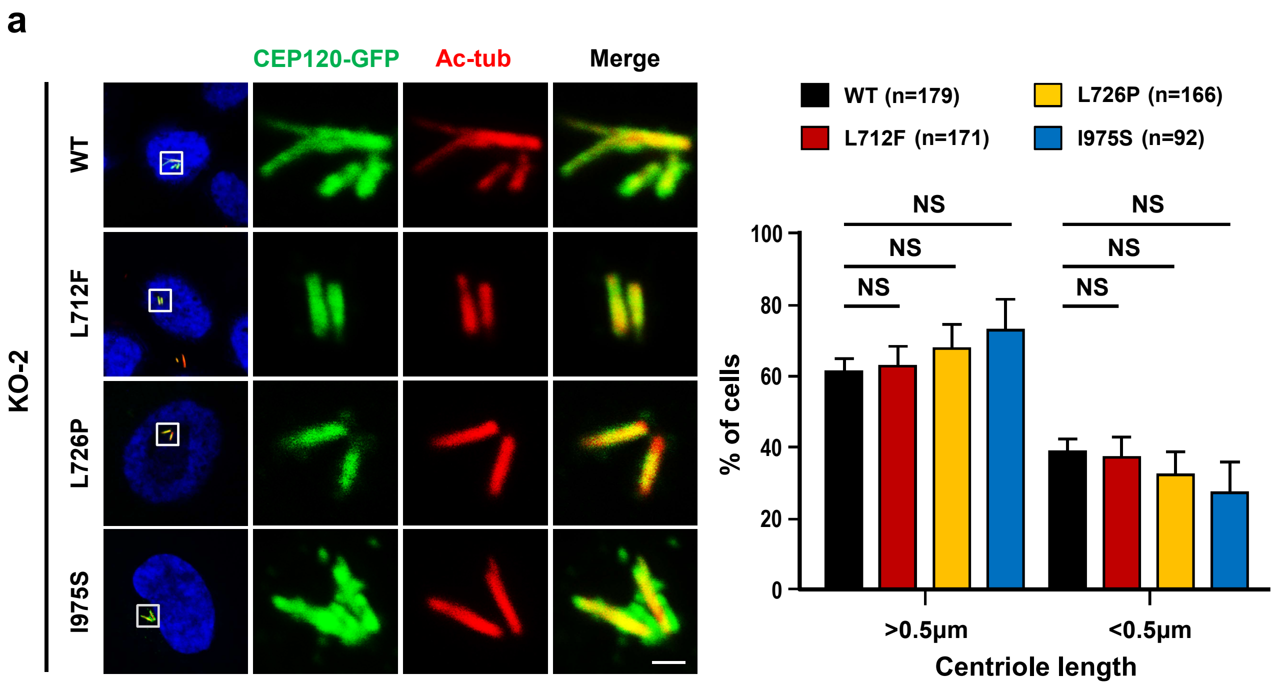

Supplementary Fig. S5. Disease-associated CEP120 mutants have no effect on centriole elongation. KO-2 cells expressing doxycycline-inducible wild-type (WT) or mutant (L712F, L726P, I975S) CEP120-GFP were synchronized at G2 and analyzed with the indicated antibodies. Histogram illustrating the percentages of cells with overly long centrioles (centriole length > 0.5 μm). Error bars represent the mean ± s.d. from pools of cells (WT, n = 179; L712F, n = 171; L726P, n = 166; I975S, n = 92) from three independent experiments. NS, not significant (two-tailed unpaired t-test). Scale bar, 1 μm.

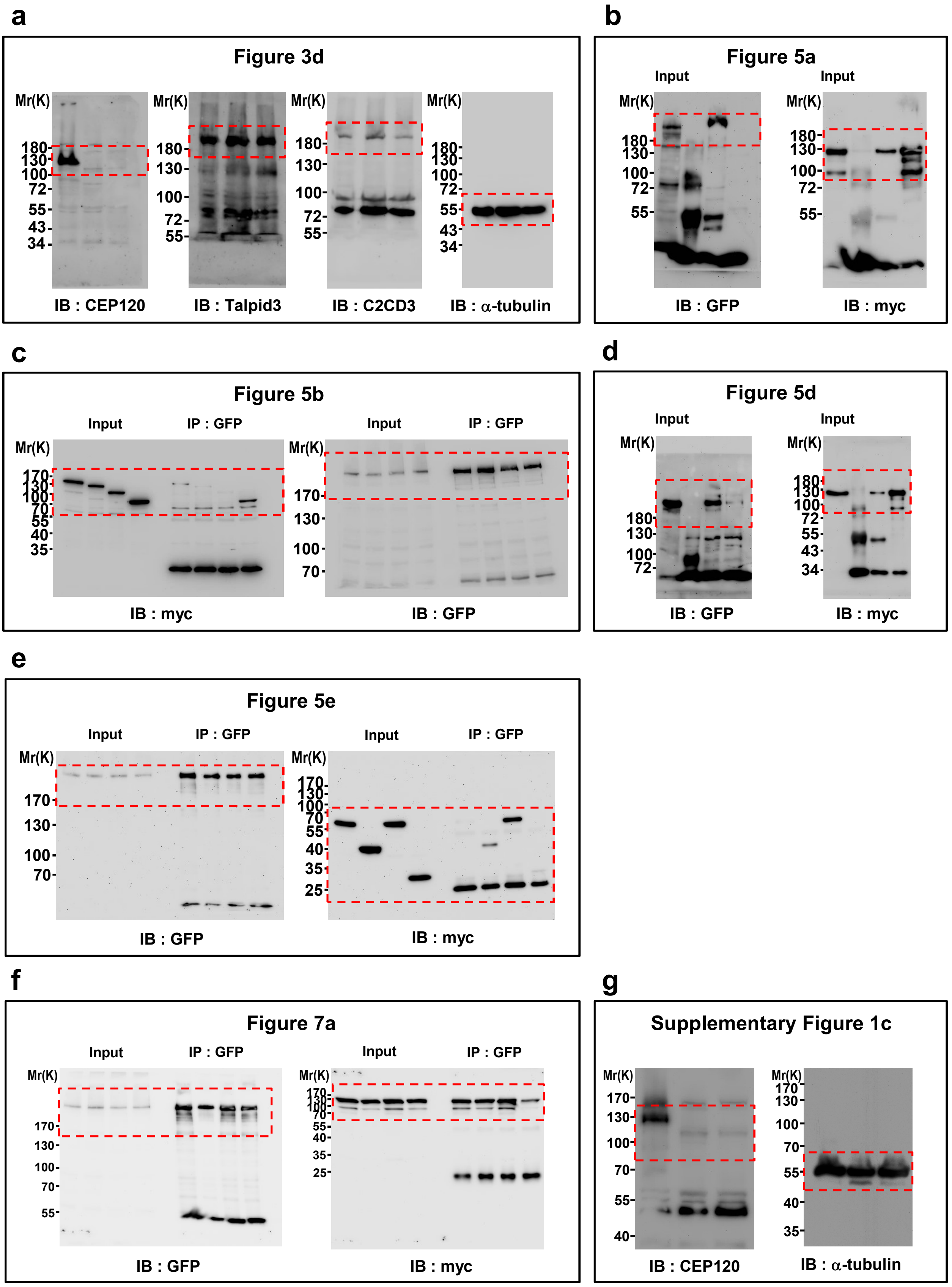
a
Figure 3d
Mr(K)
180
130
100
72
55
43
34
IB : CEP120
IB : Talpid3
IB : C2CD3
IB : α-tubulin
b
Figure 5a
Input
IB : GFP
IB : myc
c
Figure 5b
Input
IP : GFP
IB : myc
IB : GFP
d
Figure 5d
Input
IB : GFP
IB : myc
e
Figure 5e
Input
IP : GFP
IB : GFP
IB : myc
f
Figure 7a
Input
IP : GFP
IB : GFP
IB : myc
g
Supplementary Figure 1c
IB : CEP120
IB : α-tubulin

Supplementary Fig. S6. Full-length blots corresponding to Figures 3, 5, 7 and S1. (a) Blots corresponding to figure 3d. The bands on the membranes close to the protein masses of CEP120, Talpid3, C2CD3, and α-tubulin were cut and aligned before exposure. (b) Blots corresponding to figure 5a. The bands on the membranes close to the protein masses of GFP-C2CD3 and CEP120-myc were cut and aligned before exposure. (c) Blots corresponding to figure 5b. The bands on the membranes close to the protein masses of GFP-C2CD3 and various truncated proteins of CEP120-myc were cut and aligned before exposure. (d) Blots corresponding to figure 5d. The bands on the membranes close to the protein masses of GFP-Talpid3 and CEP120-myc were cut and aligned before exposure. (e) Blots corresponding to figure 5e. The bands on the membranes close to the protein masses of GFP-Talpid3 and various truncated proteins of CEP120-myc were cut and aligned before exposure. (f) Blots corresponding to figure 7a. The bands on the membranes close to the protein masses of GFP-C2CD3 and various CEP120-myc mutants were cut and aligned before exposure. (g) Blots corresponding to supplementary figure 1c. The bands on the membranes close to the protein masses of CEP120 and α-tubulin were cut and aligned before exposure.
